# Optimization of the Ultrasound Operating Conditions for Extraction and Quantification of Fructooligosaccharides from Garlic (*Allium sativum* L.) via High-Performance Liquid Chromatography with Refractive Index Detector

**DOI:** 10.3390/molecules27196388

**Published:** 2022-09-27

**Authors:** Muhammad Abdul Rahim, Adeela Yasmin, Muhammad Imran, Mahr Un Nisa, Waseem Khalid, Tuba Esatbeyoglu, Sameh A. Korma

**Affiliations:** 1 Department of Food Science, Faculty of Life Sciences, Government College University, Faisalabad 38000, Pakistan; 2 Department of Nutritional Sciences, Faculty of Medical Sciences, Government College University, Faisalabad 38000, Pakistan; 3 Department of Food Development and Food Quality, Institute of Food Science and Human Nutrition, Gottfried Wilhelm Leibniz University Hannover, Am Kleinen Felde 30, 30167 Hannover, Germany; 4 Department of Food Science, Faculty of Agriculture, Zagazig University, Zagazig 44519, Egypt

**Keywords:** garlic (*Allium sativum* L.), FOS, prebiotics, UAE, RSM, HPLC-RID, spice, detection quantification

## Abstract

Dietary interventions have captured the attention of nutritionists due to their health-promoting aspects, in addition to medications. In this connection, supplementation of nutraceuticals is considered as a rational approach to alleviating various metabolic disorders. Among novel strategies, prebiotic-supplemented foods are an encouraging trend in addressing the issue. In the present investigation, prebiotic fructooligosaccharides (FOS) were extracted from garlic (*Allium sativum* L.) powder using ultrasound-assisted extraction (UAE). The response surface methodology (RSM) was used to optimize the independent sonication variables, i.e., extraction temperature (ET, 80, 90, and 100 °C), amplitude level (AL, 70, 80, and 90%) and sonication time (ST, 10, 15 and 20 min). The maximum FOS yield (6.23 ± 0.52%) was obtained at sonication conditions of ET (80 °C), AL (80%) and ST (10 min), while the minimum yield of FOS was obtained at high operating temperatures and time. The optimized FOS yield (7.19%) was obtained at ET (80 °C), AL (73%) and ST (15 min) after model validation. The influence of sonication parameters, i.e., ET, AL and ST, on FOS yield was evaluated by varying their coded levels from −1 to +1, respectively, for each independent variable. High-performance liquid chromatography with refractive index detector (HPLC-RID) detection and quantification indicated that sucrose was present in high amounts (2.06 ± 0.10 g/100 g) followed by fructose and glucose. Total FOS fractions which included nystose present in maximum concentration (526 ± 14.7 mg/100 g), followed by 1-kestose (428 ± 19.5 mg/100 g) and fructosylnystoses (195 ± 6.89 mg/100 g). Conclusively, garlic is a good source of potential prebiotics FOS and they can be extracted using optimized sonication parameters using ultrasound-assisted techniques with maximum yield percentage.

## 1. Introduction

Consumers are cautious about their food in recent times as poor nutritional habits such as more intake of saturated fatty acids and sugar contents and low intake of long-chain polyunsaturated fatty acids, vitamins, minerals and dietary fibers result in heart diseases, metabolic syndrome, chronic anxiety disorders, inflammation and various other maladies both in developed and developing countries. People need those functional foods that not only fulfil the nutritional requirements but also provide bioactive compounds that help in maintaining good health and longevity [1]. Therefore, the utilization of functional ingredients is important to provide the health benefits that ultimately reduce these risk factors due to poor nutritional intake. In this regard, many food industries are more interested in the production of fortified food products using different functional ingredients than actual food. The important functional ingredients in the human diet are prebiotics, probiotics, polyphenols, fatty acids and vitamins [2]. Among these functional ingredients, prebiotics plays an effective role in intestinal health by selectively stimulating the growth and activity of bacteria in the bowel [3]. Prebiotics act as feed for probiotics bacteria and other beneficial microbiota in the small intestine. It produces more health benefits by modulating intestinal microbiota as compared to other techniques such as drug therapy, aging, disease and antibiotics. It helps to promote certain microbial species, which are not present in the gut, for gaining better health benefits [4].

The prebiotics concerned are present in vegetables, roots and tuber crops. Among roots, garlic is an excellent source of natural prebiotics in the form of fructooligosaccharides (FOS). FOS are a diverse group of carbohydrates including fructose residues as prime monomers [5]. FOS, also synonymously called as oligofructose or oligofructan, are oligosaccharide fructans. Short-chain fructooligosaccharides (scFOS or FOS) are a combination of 1F-(1-β-fructofuranosyl) n-1 sucrose oligomers, where n varies from two to four [6]. In nature, they are sucrose molecules (glucose–fructose disaccharide) to which one or more extra fructose units are connected by β, 2-1glycosidic linkages The individual components of FOS contain GF_2_ (α-D-glucopyronoside-(1,2)-β-D-fructofuranosyl-(1,2)-β-D-fructofuranosyl or kestose), GF_3_ (α-D-glucopyronoside-(1,2)-β-Dfructofuranosyl-(1,2)-β-D-fructofuranosyl-(1,2)-β-D-fructofuranosyl-(1,2)-β-Dfructofuranosyl (nystose) and 1F-fructofuranosyl-nystose (GF_4_) [7]. Bioactive compounds 1-kestose (GF_2_), nystose (GF_3_), quercetin, kaempferol and fructosylnystose (GF_4_) have been reported in garlic samples [8,9]. FOS are also responsible for many functions in the human body such as the consumption of non-digestible oligosaccharides that increase gastrointestinal metabolism, improve the activity of bifidobacteria in the large intestine and act as an essential nutrient that must be present in the diet to reduce the risk of heart diseases and maintain gut health. Additionally, FOS also act as an antimicrobial agent, antioxidant, hypoglycemic index and hepatoprotective compounds, lowering alanine aminotransferase and mineral absorption to maintain bone homeostasis and maintain lipid profile levels in the human body [10]. Femia et al. [11] reported that FOS could reduce colonic epithelial cell proliferation in the colon and reduce the number of pills. They also help to reduce plasma cholesterol and hypertriglyceridemia and maintain colon health and gut microflora [12]. This dietary fiber can also help to reduce the effect of hypertriglyceridemia and decrease glucose intolerance in the colon [13]. Furthermore, FOS can also be used as a sweetener in the form of sucrose as it contains low calories and is rich in fiber [10,13].

Prebiotics are commonly obtained through three main techniques, i.e., microbiological synthesis, enzymatic degradation of polysaccharides and isolation from natural resources. These are naturally present in vegetables, roots and tuber crops. Among roots, garlic has been recognized as an excellent source of natural prebiotics in the form of FOS, comprising 70–80% of its total dry matter. Commercial extraction and quantification methods have been employed for the determination of prebiotic oligosaccharides from different vegetables and fruits such as enzymatic extraction, electrophoresis and ion exchange chromatography; most of these methods are technically complicated, require many laborious steps, with many impurities, and are time-consuming and expensive [10,14,15,16]. In addition, previous research has elaborated that the extraction yield of prebiotics FOS can be increased by 25% using ultrasound-assisted extraction (UAE) technology when compared to traditional enzymatic and solvent extraction methods. High-performance liquid chromatography (HPLC) is the method of choice among the chromatographic techniques for the quantification and detection of FOS and their other structural components [15,17,18]. Optimization of the methods for natural product extraction and quantification may reduce the cost and time consumption with a higher yield. In this connection, the present research aimed to optimize the ultrasound operating conditions and check their impact on FOS extraction from garlic (*Allium sativum* L.) powder using response surface methodology (RSM) and to determine their main sugar contents of FOS by high-performance liquid chromatography with refractive index detection (HPLC-RID) for the first time in Pakistan.

## 2. Results and Discussion

### 2.1. Model Fitting and Extraction Yield of Fructooligosaccharides (FOS)

In order to optimize extraction conditions, the combined impact of independent variables (extraction temperatures (ET) 80–100 °C, amplitude level (AL) 70–90% and sonication time (ST) 10–20 min on the extraction of FOS, experiments were performed for different combinations of the independent variables using statistically designed experiments, and the results have been described in Table 1. The total number of the experiment was 16-run to determine their optimum levels. In this study, the highest yield of FOS in garlic powder was obtained at 6.23 ± 0.52% at ET (80 °C), AL (80%), and ST (10 min). The minimum response value in experimental samples was estimated at 4.55 ± 0.40% at ET (100 °C), AL (80%) and ST (20 min). Further, the FOS yield was significantly improved by reducing the ET and ST. The predicted extraction of FOS from garlic powder with the combinations of independent variables such as ET (°C), AL (%) and ST (min) as defined by the regression model were found in the range of 5.75 ± 0.44% to 7.19 ± 0.57% (Table 1). 

There is very limited published data that provides information to support the extraction of FOS from garlic powder using optimized operating conditions of ultrasound-assisted extraction (UAE). The predicted values of FOS were compared with experimental values in order to evaluate the validity of the model. Table 2 indicates the analysis of variance (ANOVA) obtained from the fitting of the experimental data and the interaction effect of ultrasonic conditions on FOS yield. The model and lack of fit *f*-value showed a significant effect on the dependent variables. The R_2_ computed for FOS was found to be 0.92. The analytical results for adjusted and predicted R_2_ values were reported as 0.80 and 0.25, respectively.

UAE is based on the propagation of mechanical waves that are capable of destroying the cell walls of the sample. It analyzes the variables involved in the production of high-value compounds and the extraction of prebiotics [19]. The FOS content in garlic powder was found to be 3.34% as described in a research study conducted by Prayogi Sunu et al. [20]. According to the literature of Campbell et al. [14], FOS content in garlic powder was 1.70%. The FOS yield depends on the concentration, temperature, solvent and treatment time. Moreover, the temperature and time comprehensively affected the yield of bioactive compounds [21]. Another research work by Heydari and Darabi Bazvand [22] revealed that the maximum extraction efficiency of vitamin C or ascorbic acid was estimated in various matrices at the lower ultrasonic time (10 s) and higher ultrasonic amplitude (100%). In another similar study, the extraction efficiency of mineral components from edible oils was increased up to 10 min and then decreased, while increasing the optimum ultrasonic bath temperature to 60 °C contributes to an increase in the yield [23]. Furthermore, the maximum yield was obtained at optimizing ultrasonic conditions such as, ultrasound time = 30 min; volume of organic solvent = 2.5 mL; salt concentration = 25% *w*/*v*; and pH = 4 [24]. According to the previous report of Rezaeepour et al. [25], a higher extraction efficiency occurs at the initial ultrasonic time range from 1 to 30 min and then decreases. A comparative study was carried out by Louie et al. [26] and found a significantly higher yield of FOS as compared to other traditional extraction methods. The improvement in yield was noticed as time and temperature decreased [10]. The highest value of yield (112 µg/mL) was determined at 25 °C for 90 min with optimum frequency [21]. The highest withdrawal rate was observed in the first few minutes, which is considered to be the most profitable period [27]. Higher FOS contents can be used in functional products to improve the activity of microbiota and may reduce the attack of pathogens on intestinal cells [28].

### 2.2. Single Factor Analysis for FOS Yield

**Figure 1:**
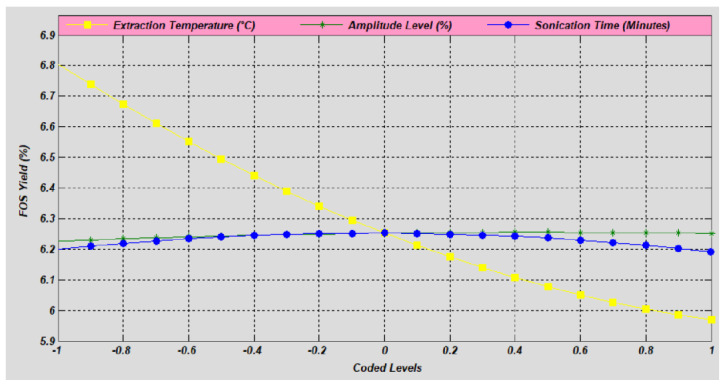
Single factor analysis of independent sonication operating conditions for FOS yield. For interpretation of the references to color in this figure legend, the reader is referred to the Web version of this article.).

The influence of ET, AL and ST on FOS yield was evaluated by varying their coded levels from −1 to +1, respectively, for each independent variable (Figure 1). The mean value of independent variables was set to rotate the model uniformly during the analysis of each individual variable of the process and response. The regression equations for the independent variables are given in Equations (1)–(3), respectively.

Regression equation for ET = (36.492) + (−0.609) ET + (0.003) ET^2^ (1)

Regression equation for AL = (4.1025) + (0.051875) AL + (−0.003) AL^2^ (2)

Regression equation for ST = (5.108) + (0.153) ST + (−0.005) ST^2^ (3)

The analysis of the single factor showed that the ET had a strong effect on the percentage of FOS yield. The FOS yield was inversely proportional to the level of ET. The FOS yield was increased by lowering the level of ET. The AL and ST level imparts minimum effect on the FOS yield (Figure 1). The regression coefficient was used to calculate the quadratic impact of independent variables. In this quadratic regression model, the regression coefficient between the independent variables and the response variables was high, indicating the best evaluation of the experimental data.

### 2.3. Mutual Interaction Effect on FOS Yield

**Figure 2:**
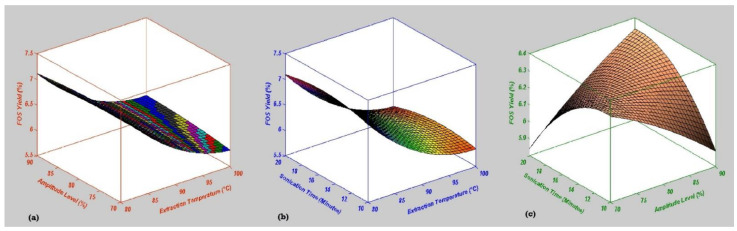
Surface graphical representation of mutual interaction effect of sonication conditions on response yield. (**a**) Extraction temperature (°C) and amplitude level (%) versus FOS yield (%), (**b**) extraction temperature (°C) and sonication time (min) versus FOS yield (%) and (**c**) amplitude level (%) and sonication time (min) versus FOS yield (%). For interpretation of the references to color in this figure legend, the reader is referred to the Web version of this article.

**Figure 3:**
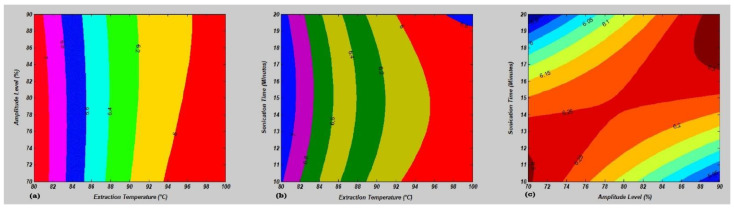
Mutual interaction analysis of sonication operating conditions in terms of contour plots for FOS yield. (**a**) Extraction temperature (°C) versus amplitude level (%), (**b**) extraction temperature (°C) versus sonication time (min) and (**c**) amplitude level (%) versus sonication time (min). For interpretation of the references to color in this figure legend, the reader is referred to the Web version of this article.

The effect of the mutual interaction on the independent variables for the yield of FOS in garlic powder was estimated by rotating two independent factors and fixing the third factor at the coded zero level. The surface and contour plots representing the mutual interaction of sonication independent variables have been shown in Figure 2 and Figure 3. The mutual interaction effect between ET and AL showed that the FOS yield was reduced by increasing the ET and AL (Figure 2a). Furthermore, the correlation between ST and ET indicated that the increase in ST and ET leads to a decline in FOS yield (Figure 2b). Moreover, the FOS yield was improved by lowering the ST and AL levels (Figure 2c). The validation of the model depends on the optimized experimental values and response yield. The Box–Behnken design (BBD) was used to optimize the operational conditions and FOS yield. Based on the above findings, the interaction between ET and AL showed the FOS yield of 7.19% at ET (80 °C), AL (73.34%) and ST (15 min) (Figure 3a). Moreover, the relation between optimized and predicted values of ET and ST indicated the FOS yield as 7.18%, at ET (80 °C), AL (80%) and ST (15.67 min) (Figure 3b). Furthermore, sonication-independent conditions for AL and ST in UAE were determined as ET (90 °C), AL (90%) and ST (19.34 min) for a 6.33% FOS yield (Figure 3a). The optimized sonication conditions for FOS yield validation were again performed with three different replications to confirm the final predicted value and response yield for future recommendations at the commercial scale for discerning food processing industries. Finally, the optimized FOS yield (7.19%) was obtained at ET (80 °C), AL (73%) and ST (15 min) after model validation.

The results of the present study are in line with the previous findings of Ahmad et al. [29]. Notably, they used extraction temperature, extraction time and liquid–solid ratio in the representative quadratic model and statistical validation of the polynomial equation was performed. The highest polysaccharide yield (11.56%) was noted at optimum conditions [29]. The mutual interaction between response and predicted values was validated by the RSM model using regression coefficient correlation [30,31]. In a similar fashion, the effect of time, temperature, volume to mass ratio and ultrasound treatment on yield was validated for individual regression coefficients [32]. Moreover, Khumpirapang et al. [33] described the strong correlation between predicted and experimental values obtained at optimal extraction conditions, and such a finding ultimately strengthens the outcomes reported in the present study.

### 2.4. Quantification of FOS by High-Performance Liquid Chromatography with Refractive Index Detector (HPLC-RID)

The quantification of FOS via HPLC-RID in garlic powder is presented in Table 3. It is obvious from the data that sucrose is present in the highest amount (2.06 ± 0.10 g/100 g), followed by fructose and glucose. On the other hand, out of total FOS fractions, nystose (GF_3_) with three glucose units was present in maximum concentration (526 ± 14.68 mg/100 g), preceded by 1-kestose (GF_2_) at 428 ± 19.45 mg/100 g and fructosylnystose (GF_4_) 195 ± 6.89 mg/100 g. The results from intra- and inter-day analysis showed good precision. The results concerning extracted FOS from garlic in the current investigation are in agreement with the observations of Król and Grzelak [34]. They categorized commercially available FOS and observed the values for individual monosaccharides containing sucrose, glucose and fructose as 3.00, 0.40 and 0.30 g/100 g, whilst nystose, kestose, and fructosylnystose were observed as 49.00, 36.00 and 12.00 g/100 g. Nevertheless, the FOS composition varies with the source, degree of polymerization as well as the method of extraction. The obtained results for FOS composition are also corroborated by the findings of Chen et al. [35], who assessed FOS powder for its modulating role in elderly men. According to their observations, HPLC analysis exposed that FOS contained various fractions i.e., sucrose, glucose, fructose, 1-kestose, nystose and fructosylnystose. Out of these individual fractions, the maximum level was noticed for 1-kestose and the minimum for fructosylnystose. The current data regarding FOS characterization are in agreement with the findings of Judprasong et al. [36]; they verified the FOS and inulin composition of numerous fruits and vegetables including garlic and established the presence of the above-mentioned fractions.

FOS hold quite a lot of characteristics that make them a desirable food additive to augment the shelf life and taste profile of food products [7]. Remarkably, FOS are one of the new functional fibers employed in the food industry that effectually increase the fiber contents of usually non-fibrous foods. Still, it is appropriate to probe the compositional parameters and degree of polymerization of FOS contents of different vegetables as it impacts the rheology of functional food products to which they can be added. Moreover, the chain length also affects the level of fermentation in the intestine and a shorter chain length is preferred [37].

Garlic has a peculiar smell due to which it is not liked by all consumers. Moreover, its pungent taste may not be appropriate for addition to some types of food products. Hence, extraction of FOS and provision in their purified form may improve their overall consumption. The extracted FOS in this study were off-white in color and almost odorless. So, they can be used as an ingredient in the preparation of many ready-to-eat food products with prebiotic properties.

## 3. Materials and Methods

### 3.1. Chemicals and Preparation of Sample

All the chemicals and HPLC grade reagents and standards were purchased from Merck (Merck KGaA, Darmstadt, Germany) and Sigma-Aldrich (Sigma-Aldrich, Tokyo, Japan). Spring garlic was purchased from the registered superstore, Punjab, Pakistan. In this study, garlic bulbs were manually separated into cloves. The undesirable components were removed from the garlic cloves. After that, approximately 100 g of samples were randomly selected, cut into small pieces of 20 to 30 mm and dried in a universal hot air oven (Memmert^®^ UN55, Memmert, Schwabach, Germany) for 12 to 24 h at 60 °C. At the end, garlic powder was prepared by grinding the dry bulb through a grinder [38].

### 3.2. Ultrasound-Assisted Extraction (UAE) of Fructooligosaccharides (FOS)

FOS were extracted from the garlic powder samples using the method elaborated by Jovanovic-Malinovska et al. [18] with some modifications. In this method, 100 g of garlic powder was dissolved in 20 mL of ethanol (96%) and prepared into a solution. The sonication apparatus (model VCX 750, Sonic and Materials, Inc., Newtown, CT, USA) was used for the extraction process. The different ratios of prepared solutions were placed in the ultrasound sonication apparatus at extraction temperatures (ET) 80–100 °C, amplitude level (AL) 70–90% and sonication time (ST) 10–20 min. After that, the solution samples were kept for 10 to 20 min to cool at room temperature (25 ± 2 °C). Then, the solution was centrifuged at a low temperature of 10 °C by adjusting the speed at 3000 rpm. After the centrifugation, 10 mL of supernatants were mixed and placed in the vacuum rotary evaporator at 50 °C until the solvent was removed. Finally, the slurry was re-suspended by adding 1.5 mL of deionized water. Whatman No. 1 filter paper was used for filtration.

### 3.3. Determination of the FOS Content by HPLC-RID

The concentration of FOS in the garlic powder sample was estimated using HPLC-RID (PerkinElmer Series 200, PerkinElmer, Shelton, CT, USA) fitted with a refractive index detector (RID-10A) and C18 column (250 mm × 4.6 mm, 5.0 μm particle size). In this study, 1 mL sample was dissolved in HPLC grade water and the solution volume was made up to 50 mL. After that, the prepared solution was kept in a shaking water bath for 5 min at 30 °C and filtered with Whatman No. 1 (pore size 25 μm). Then, 10 μL of the aliquot sample was injected. The mobile phase was HPLC grade water. The flow rate was adjusted to 1 mL/minutes and the amounts of 1-kestose (GF_2_), nystose (GF_3_), fructosylnystose (GF_4_) and total (FOS) were performed by their respective standards of fructose, glucose, sucrose and FOS (Sigma-Aldrich, St. Louis, MO, USA). The precision or accuracy of the presented method was assessed through the intra- and inter-day repeatability of the method of respective standards. The intra-day repeatability study was performed through injection of standard solution six times in one day followed by calculation of the relative standard deviation. Furthermore, inter-day repeatability was assessed by analyzing the same standard solution once a day over a three-day period [39].

### 3.4. Experimental Design and Statistical Analysis

The obtained data for garlic powder were subjected to statistical analysis, and the level of significance was determined at 5% (*p* ≤ 0.05) using the software Design Expert^®^ (version 11.1.2.0, E Hennepin Ave, Minneapolis, MN, USA) and MathWorks Matlab^®^ (version 7.5.0.338; R2007a, Natick, MA, USA) software according to the method described in Montgomery [40]. In addition to this, response surface methodology (RSM) was performed to estimate the optimized values of independent variables on which maximum dependent response was obtained using the Box–Behnken design (BBD). Analysis of variance (ANOVA) was employed to check the ampleness of the model. The modeling was started with a quadratic model including linear effects, interaction effects and quadratic effects. Significant terms in the model for dependent variables were found by ANOVA and significance was assessed by the F-statistic intended from the data. The results were evaluated with various descriptive statistical analyses such as the sum of squares (SS), degree of freedom (DF); and mean square (MS). After fitting the value to the model, the generated values were used for contour and surface plots. Table 4 presents the coded and actual values of experimental treatments. The three independent variables at three levels and results obtained for each response were used to evaluate the BBD of RSM explained by non-linear Equations (4)–(6), respectively.

Y = β_0_ + β_1_ (ET) + β_2_ (AL) + β_3_ (ST) + β_1_ × β_2_ (ET × AL) + β_1_ × β_3_ (ET × ST) + β_2_ × β_3_ (AL × ST) + β_1_ (T)^2^ + β_2_ (AL)^2^ + β_3_ (ST)^2^ (4)

The regression equation in terms of coded and actual factors is given below:

Y_1_ = + 4.25 − 0.63 β_1_ + 0.019 β_2_ − 0.005 β_3_ + 0.060 β_1_ × β_2_ − 0.037 β_1_ × β_3_ + 0.23 β_2_ × β_3_ + 0.30 β_12_ − 0.031 β_22_ − 0.13 β_3_ (5)

Y_2_ = + 39.96 − 0.64 ET − 0.070 AL − 0.14 ST + 0.006 ET × AL − 0.007 ET × ST + 0.004 AL × ST + 0.003 ET^2^ − 0.003 AL^2^ − 0.005 ST^2^ (6)

where Y, Y_1_, Y_2_ = Dependent variables; β_0_ = Intercept; β_1_ = Extraction Temperature (ET); β_2_ = Amplitude Level (AL); β_3_ = Sonication Time (ST); β_1_ to β_3_ = Regression coefficients; ET, AL, ST = Independent variables.

## 4. Conclusions

This study has provided detailed information regarding the extraction of fructooligosaccharides (FOS) from the local garlic variety consumed in Pakistan. It may be concluded that garlic may be one of the major sources of FOS and the maximum yield of fructooligosaccharides (FOS) from garlic can be obtained by using the optimized conditions of ultrasound green technology. Moreover, the HPLC-RID quantification revealed the presence of 1-kestose (GF_2_), nystose (GF_3_) and fructosylnystose (GF_4_) in higher concentrations as individual sugar fractions. The results may provide a good basis for optimized extraction parameters as well as the composition of FOS in garlic. Furthermore, the present study also recommended that the extracted FOS may be explored as a functional food ingredient to formulate prebiotics-supplemented food products. Additionally, long-term storage quality and biological evaluation of such FOS-fortified food products should be considered in future studies.

## Figures and Tables

**Table 1:** Extraction yield of FOS in garlic as analyzed using Box–Behnken design (BBD) after ultrasound treatment.

Sonication Run	Independent Variables						Response Variable	
	Coded (ET)	Coded (AL)	Coded (ST)	ET (°C)	AL (%)	ST (min)	FOS Yield (%)	
							Experimental Value	Predicted Value
1	+1	−1	0	100	70	15	4.76 ± 0.42 ^h^	5.82 ± 0.48 ^e^
2(C_1_)	0	0	0	90	80	15	5.25 ± 0.47 ^fg^	6.25 ± 0.53 ^c^
3	−1	0	+1	80	80	20	5.95 ± 0.49 ^de^	7.08 ± 0.55 ^ab^
4	−1	−1	0	80	70	15	6.06 ± 0.50 ^d^	7.19 ± 0.57 ^a^
5	+1	+1	0	100	90	15	5.13 ± 0.45 ^g^	5.97 ± 0.50 ^de^
6	0	+1	−1	90	90	10	4.62 ± 0.41 ^hi^	5.88 ± 0.49 ^e^
7	−1	0	−1	80	80	10	6.23 ± 0.52 ^c^	7.02 ± 0.53 ^b^
8	0	−1	+1	90	70	20	5.11 ± 0.44 ^g^	5.84 ± 0.48 ^e^
9(C_2_)	0	0	0	90	80	15	5.27 ± 0.48 ^fg^	6.25 ± 0.53 ^c^
10	−1	+1	0	80	90	15	6.17 ± 0.51 ^cd^	7.11 ± 0.56 ^ab^
11(C_3_)	0	0	0	90	80	15	5.23 ± 0.46 ^fg^	6.25 ± 0.53 ^c^
12	+1	0	+1	100	80	20	4.55 ± 0.40 ^i^	5.75 ± 0.44 ^ef^
13	+1	0	−1	100	80	10	4.98 ± 0.43 ^gh^	5.84 ± 0.48 ^e^
14	0	−1	−1	90	70	10	5.24 ± 0.47 ^fg^	6.30 ± 0.54 ^bc^
15	0	+1	+1	90	90	20	5.41 ± 0.48 ^f^	6.33 ± 0.54 ^bc^
16(C_4_)	0	0	0	90	80	15	5.26 ± 0.45 ^fg^	6.25 ± 0.53 ^c^

C_1_, C_2_, C_3_ and C_4_ FOS sonication conditions are set at center points of the Box–Behnken design (BBD); ^a–i^ Means with different superscripts represent the change in FOS yield; ET, extraction temperature; AL, amplitude level; ST, sonication time; FOS, fructooligosaccharides.

**Table 2:** Analysis of variance (ANOVA) for the quadratic model of FOS yield.

Source of Variation	SS	DF	MS	*f*-Value	*p*-Value
Model	3.81	9	0.42	7.84	0.01
ET	3.14	1	3.14	58.18	0.003
AL	0.06	1	0.004	0.052	0.82
ST	0.04	1	0.002	0.003	0.95
ET × AL	0.01	1	0.014	0.27	0.62
ET × ST	0.05	1	0.003	0.10	0.75
AL × ST	0.21	1	0.21	3.84	0.09
ET^2^	0.37	1	0.37	6.84	0.03
AL^2^	0.01	1	0.0005	0.072	0.79
ST^2^	0.06	1	0.066	1.23	0.30
Residual	0.31	6	0.054	–	–
Lack of Fit	0.34	3	0.11	368.77	0.82
Pure Error	0.07	3	0.001	–	–
Total	4.13	16	–	–	–

ET, extraction temperature; AL, amplitude level; ST, sonication time; SS, the sum of squares; DF, degree of freedom; MS, mean square. Level of significance: *p* ≤ 0.05.

**Table 3:** Quantification of individual fractions (mono- and oligosaccharides) of FOS contents of garlic powder by HPLC-RID.

**Sugar**	**Concentration (g/100 g)**
Fructose	1.30 ± 0.09
Glucose	0.30 ± 0.02
Sucrose	2.06 ± 0.10
**Fructooligosaccharides**	**Concentration (mg/100 g)**
1-Kestose (GF_2_)	428 ± 19.45
Nystose (GF_3_)	526 ± 14.68
Fructosylnystose (GF_4_)	195 ± 6.89
Total (FOS)	1149 ± 22.35

Values expressed as means ± standard deviation.

**Table 4:** Coded and actual levels of independent sonication variables for optimization of fructooligosaccharides yield determined by BBD of response surface methodology (RSM).

Independent Variables	Units	Coded Levels		
		−1 (Low)	0 (Medium)	+1 (High)
Extraction temperature (ET)	°C	80	90	100
Amplitude level (AL)	%	70	80	90
Sonication time (ST)	min	10	15	20

## Data Availability

Data are available from the authors upon request.
